# Evolution of JAK-STAT Pathway Components: Mechanisms and Role in Immune System Development

**DOI:** 10.1371/journal.pone.0032777

**Published:** 2012-03-07

**Authors:** Clifford Liongue, Lynda A. O'Sullivan, Monique C. Trengove, Alister C. Ward

**Affiliations:** 1 School of Medicine, Deakin University, Victoria, Australia; 2 Strategic Research Centre in Molecular & Medical Research, Deakin University, Victoria, Australia; 3 School of Life & Environmental Sciences, Deakin University, Victoria, Australia; National Cancer Institute, United States of America

## Abstract

**Background:**

Lying downstream of a myriad of cytokine receptors, the Janus kinase (JAK) – Signal transducer and activator of transcription (STAT) pathway is pivotal for the development and function of the immune system, with additional important roles in other biological systems. To gain further insight into immune system evolution, we have performed a comprehensive bioinformatic analysis of the JAK-STAT pathway components, including the key negative regulators of this pathway, the SH2-domain containing tyrosine phosphatase (SHP), Protein inhibitors against Stats (PIAS), and Suppressor of cytokine signaling (SOCS) proteins across a diverse range of organisms.

**Results:**

Our analysis has demonstrated significant expansion of JAK-STAT pathway components co-incident with the emergence of adaptive immunity, with whole genome duplication being the principal mechanism for generating this additional diversity. In contrast, expansion of upstream cytokine receptors appears to be a pivotal driver for the differential diversification of specific pathway components.

**Conclusion:**

Diversification of JAK-STAT pathway components during early vertebrate development occurred concurrently with a major expansion of upstream cytokine receptors and two rounds of whole genome duplications. This produced an intricate cell-cell communication system that has made a significant contribution to the evolution of the immune system, particularly the emergence of adaptive immunity.

## Introduction

Cytokines are secreted polypeptides that mediate specific cell-cell communication essential for the development and regulation of a range of cell types, in particular those of the immune and hematopoietic systems [1]. For example, interleukin 2 is essential for the generation of lymphocytes and NK cells [2], lambda interferons play key anti-viral roles [3], while granulocyte colony-stimulating factor contributes to neutrophil differentiation and survival, as well as facilitating hematopoietic stem cell mobilization [4]. Cytokines act via specific cytokine receptor complexes expressed on the surface of target cells. Receptor ligation mediates conformational changes in the complex that initiate intracellular signaling via associated tyrosine kinases, principally members of the Janus kinase (JAK) family [5]. These activate latent Signal transducer and activators of transcription (STAT) transcription factors that induce the expression of specific sets of genes to facilitate the appropriate cellular responses [6], [7]. Differential engagement of specific JAK-STAT pathway components produces the requisite, and often exquisitely specific, response from each cytokine receptor complex within the relevant cellular context. An important part of this system is the presence of multiple regulatory mechanisms for extinguishing JAK-STAT signaling, which can lead to various pathologies if left unchecked. These negative regulators include specific members of the SH2-domain containing tyrosine phosphatase (SHP), Protein inhibitors against Stats (PIAS), and Suppressor of cytokine signaling (SOCS) families [8]. Understanding how such a complicated signaling system has developed, and how this relates to immune system evolution, remains an important challenge.

The JAK family shares a common structure, including an N-terminal FERM domain that is involved in protein-protein interactions with specific cytokine receptors with which they are often pre-associated, an SH2-like domain, a regulatory dual –kinase (JH2) domain and a C-terminal tyrosine kinase (JH1) domain [9], [10]. Conformational changes in the receptor complex lead to the initiation of intracellular signaling via auto- and trans-phosphorylation of the associated JAKs and subsequent phosphorylation of cytokine receptor tyrosine residues [11], [12]. These phosphotyrosines then act as docking sites for various signaling proteins, including members of the STAT family of transcription factors [13]. These share the variably conserved N-terminal, coiled-coil, DNA binding, SH3 linker, and SH2 domains, followed by the least conserved C-terminal region that is responsible for transactivation [10], [14], [15]. Once docked, STAT proteins are subsequently phosphorylated by JAKs on conserved tyrosines to permit formation of activated STAT homo- or hetero-dimers via intermolecular SH2-phosphotyrosine interactions. These dimers translocate to the nucleus where they bind to specific promoter sequences, to facilitate transcription of genes necessary to mediate the relevant cellular responses [16], [17].

STATs also induce the transcription of genes encoding the SOCS family of negative regulators [8]. SOCS proteins consist of a divergent N-terminal domain, a central SH2 domain responsible for binding to specific target proteins, and a C-terminal SOCS box domain that interacts with proteasomal components [18]. SOCS proteins suppress signaling by directly blocking JAK activity, competing for docking sites on the receptor complex or targeting signaling components for degradation [8]. In addition, there are latent cytosolic proteins that negatively control the JAK-STAT pathway, principally the SHP and PIAS proteins [8]. SHP proteins possess tandem N-terminal SH2 domains that bind specifically to key substrates, a central tyrosine phosphatase domain, and a divergent C-terminal region, which contains several tyrosine residues that serve as docking sites for other signaling proteins when phosphorylated [19], [20]. PIAS proteins, on the other hand, consist of an N-terminal SAP domain, followed by a PINIT motif, a RING finger-like zinc binding domain (RLD), an acidic domain (AD), and a divergent C-terminal serine/threonine (S/T)-rich region in all members except PIASy [21].

The most primitive canonical JAK-STAT signaling pathway, consisting of a single JAK-STAT module induced by an upstream cytokine receptor and regulated by SOCS, SHP, and PIAS proteins, is found in extant invertebrates. For example, the fruit fly (*Drosophila melanogaster*) possesses a clearly discernible cytokine receptor (Domeless), along with single JAK (hopscotch), STAT (marelle/STAT92E), SHP, and PIAS proteins as well as three SOCS proteins [22]. In insects the JAK-STAT pathway contributes to anti-viral and anti-bacterial response [23], [24], [25], [26], as well as the generation of the leukocyte-like hemocytes [27], [28]. However, the pleiotropic nature of JAK-STAT signaling is manifested in a diverse range of other roles in development and maintenance, including cell fate determination, brain development, cardiogenesis, and intestinal stem cell proliferation [29], [30], [31], [32].

The JAK-STAT signaling pathway of invertebrates has been expanded upon in mammals to four JAK, seven STAT, three SHP, four PIAS, and eight SOCS family members to service over 50 cytokine and other receptors, the majority with roles in immunity and hematopoiesis but others participating in other important roles [18], [33], [34]. Perturbation of the mammalian JAK-STAT pathway often leads to immunological and hematopoietic diseases as well as various cancers. Disruption of relevant JAK-STAT signaling components generally leads to a compromised immune system, such as JAK3 mutations contributing to severe combined immune deficiency and STAT1 mutations increasing susceptibility to mycobacterial infections [7]. In contrast, aberrant activation of JAK-STAT components contributes to proliferative disorders and malignancies [35]. For example, specific JAK2 mutations play a major role in a range of myeloproliferative disorders, namely polycythaemia vera, essential thombocytosis, and primary myelofibrosis, which result in excessive expansion of erythrocytes, thrombocytes, and granulocytes respectively [36]. Similarly, TEL-JAK2 fusions contribute to leukemia [35], whilst aberrant activation of STAT3 increases ovarian cancer motility [37].

The intracellular JAK, STAT, SHP, PIAS, and SOCS signaling pathway has expanded from 7 components in fruit fly to 26 components in mammals. Many of the functions appear conserved in other vertebrates, including zebrafish [38], [39], [40], [41]. Over this same time period the immune system has increased greatly in complexity. This study has investigated the differential evolution of JAK-STAT pathway components since the formation of the canonical, cytokine receptor-regulated JAK-STAT signaling pathway, providing new insights into the process, with implications for immune system evolution.

## Results

To gain insight into JAK-STAT pathway evolution, a comprehensive bioinformatic strategy was employed to identify and characterize JAK, STAT, SHP, PIAS, and SOCS genes from a range of relevant organisms (File S1).

### *JAK* evolution

**Figure 1:**
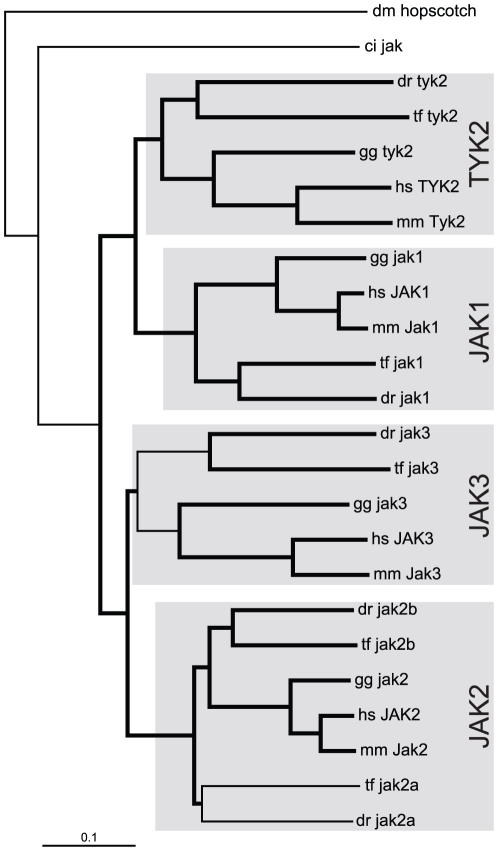
Phylogenetic analysis of *JAK* family members. Phylogenetic analysis of JAK protein sequences using the Neighborhood-Joining algorithm, with bootstrap values above 80% (of 1000 replicates) indicated in bold. Species used for phylogenetic analysis are indicated: fruit fly (dm), sea squirt (ci), zebrafish (dr), green pufferfish (tf), Japanese pufferfish (tr), chicken (gg), mouse (mm), and human (hs).

**Figure 2:**
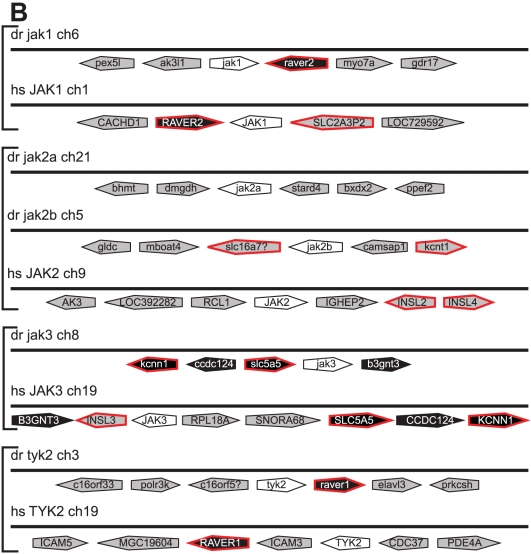
Synteny analysis of *JAK* family members. Synteny analysis of the relevant zebrafish and human JAK gene loci (white), indicating adjacent genes in their respective orientations. Genes showing conserved synteny between zebrafish and human are in black, genes which have homologues displaying synteny with other family members in humans or zebrafish edged in red and non-syntenic genes in grey.

A single *JAK* homologue has been found in both fruit fly (*hopscotch*) [42] and sea squirt (*Ciona intestinalis*) (*jak*) [43]. Mammals possess four family members (*JAK1*, *JAK2*, *JAK3*, *TYK2*) [11], with previous studies identifying a *JAK1* orthologue (*jak1*) [44] and two *JAK2* paralogs (*jak2a*, *jak2b*) [45] in zebrafish (*Danio rerio*). Bioinformatic approaches were used to complete the partial zebrafish *jak2b* sequence as well as identify two additional *JAK* family members in this organism, the expression of which were confirmed by RT-PCR (Table S1). Phylogenetic analysis (Figure 1) identified these as orthologs for *JAK3* and *TYK2*, assignments that were supported by conserved synteny data (Figure 2), including multiple genes in the case of *jak3* (*B3GNT3*, *SLC5A5*, *CCDC124*, *KCNN1*), or a single gene in the case of *tyk2* (*RAVER1*). Comparative analysis of the green pufferfish (*Tetraodon fluviatilis*) genome revealed the presence of an identical set of *JAK* genes, including both *jak2a* and *jak2b*. In contrast, chicken (*Gallus gallus*) was found to possess the same *JAK* complement as mammals, including a single *jak2*, providing evidence that there has been duplication of this gene specifically in teleosts. Phylogenetic analyses also indicated a closer evolutionary relationship between two gene pairs, the *JAK1* and *TYK2* pair and the *JAK2* and *JAK3* pair. This was also supported by conserved syntenic relationships with *RAVER*-related genes for *JAK1* and *TYK2*, as well as *INSL*- and *KCNN*-related genes for *JAK2* and *JAK3*, while association with *SLC*-related genes for *JAK1*, *JAK2*, and *JAK3* is consistent with a common evolutionary ancestry. All vertebrate JAK proteins possessed equivalent JH1-7 domains that were conserved across all members [11], with several residues, including a dityrosine within the kinase domain, being wholly conserved (File S2). Intron/exon structures were identical across all *JAK* genes with the exception of zebrafish *jak2a*, which possesses an additional exon that encodes its leader sequence.

### *STAT* evolution

**Figure 3:**
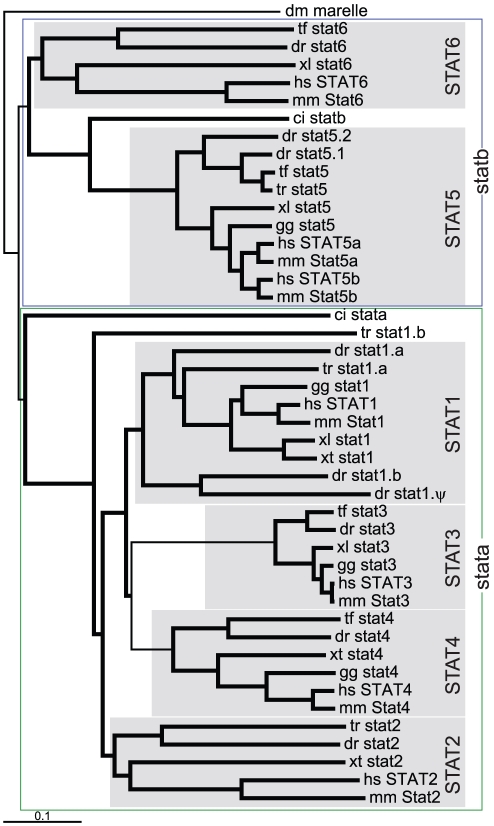
Phylogenetic analysis of *STAT* family members. Phylogenetic analysis of STAT family members as described for Figure 1, with the inclusion of genes from African clawed frog (xl) and Western clawed frog (xt).

Fruit fly possesses a single *STAT* gene (*marelle*) [46], whilst sea squirt has two *STAT* genes (*stata*, *statb*) [43]. In contrast, there are seven *STAT* members in mammals (*STAT1*, *STAT2*, *STAT3*, *STAT4*, *STAT5A*, *STAT5B*, *STAT6*), which exist in three clusters: *STAT1* and *STAT4* lying adjacent, *STAT3*, *STAT5A* and *STAT5B* lying adjacent, with *STAT2* and *STAT6* located proximally on the same chromosome [11]. Previous work in zebrafish has identified single *stat1* and *stat3* genes [47], as well as duplicate *stat5* genes, *stat5.1* and *stat5.2*, that were derived independently of the *STAT5A* and *STAT5B* duplication in mammals [48]. *In silico* analysis revealed the presence of five additional *STAT* sequences in zebrafish, the expression of which were confirmed by RT-PCR (Table S1). Phylogenetic analysis identified one of these as an additional *STAT1* paralog, designated *stat1b* (with the previously described zebrafish *stat1* [47] renamed *stat1a*), one as a *stat1* pseudogene (*stat1. Ψ*), as well as single teleost *stat2*, *stat4* and *stat6* orthologs (Figure 3).

**Figure 4:**
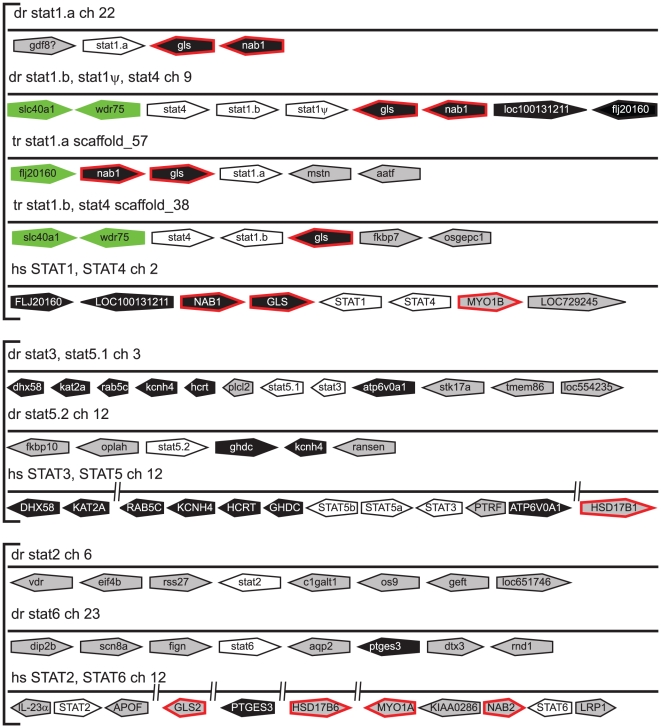
Synteny analysis of *STAT* family members. Conserved synteny analysis of STAT loci as described in Figure 2, with the addition of genes that displayed synteny between zebrafish and Japanese pufferfish being shown in green.

The STAT gene designations were generally well-supported by conserved synteny (Figure 4). For example, teleost *stat1b* and *stat4* lay adjacent, like mammalian *STAT1* and *STAT4*, with further conserved synteny of several adjacent genes (*GLS*, *NAB1*, *LOC100131221*, *FLJ20160*). The teleost-specific *stat1a* genes were also flanked by paralogs to several of these (*GLS*, *NAB1*), while the *stat1* pseudogene lay immediately downstream of *stat1b*, suggestive of a local duplication event. Only a single gene (*PTGES3*) showed conserved synteny with *stat6* while no synteny conservation was evident for *stat2*. However, the identity of the latter was confirmed by the presence of a KYLK motif in the encoded protein that is unique to STAT2 [14], as well as a similar splice structure (File S3).

Conserved synteny was also evident between various *STAT* gene clusters, such as the *GLS-*, *MYO1-* and *NAB*-related genes between the *STAT1/STAT4* cluster and *STAT2/STAT6* cluster as well as *HSD17B*-related genes between the *STAT3/STAT5* cluster and *STAT2/STAT6* cluster. Phylogenetic analysis suggested two distinct STAT sub-families, one containing vertebrate *STAT1*, *STAT2*, *STAT3* and *STAT4* along with sea squirt *stata*, and one containing vertebrate *STAT5* and *STAT6* along with sea squirt *statb*. This distinction was also supported by the alternative splice structure in the region encoding the coiled-coil and DNA binding domains between these two sub-families (File S3).

### *SHP* evolution

**Figure 5:**
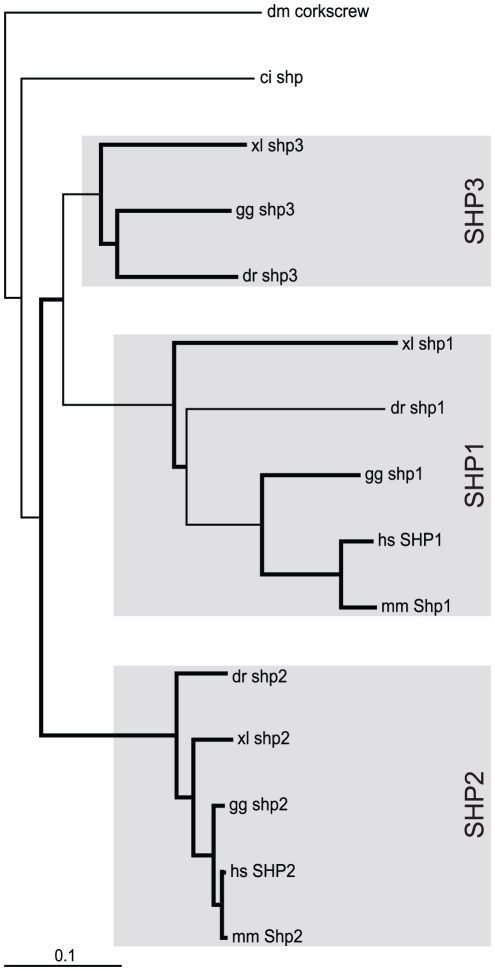
Phylogenetic analysis of *SHP* family members. Phylogenetic analysis of *SHP* family members as described for Figure 3.

**Figure 6:**
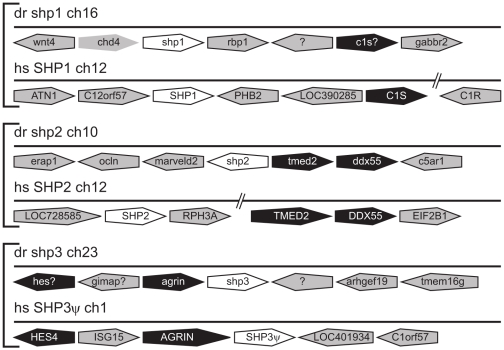
Synteny analysis of *SHP* family members. Syntenic analysis of *SHP* family members as described for Figure 4.

Single *SHP* homologues have been identified in fruit fly (*corkscrew*) [49] and in sea squirt (*shp*) [43], whilst mammals possess two family members (*SHP1*, *SHP2*) [50]. Analysis of vertebrate genomes revealed three *SHP* homologues in zebrafish, with expression confirmed by RT-PCR and the presence of corresponding EST sequences in each case (Table S1), as well as related genes in several tetrapods, including chicken and toad. Phylogenetic analysis identified two of these as orthologs of mammalian *SHP1* and *SHP2* (Figure 5), confirmed by conserved synteny relationships for *shp1* (*C1S*) and *shp2* (*TMED2*, *DDX55*) (Figure 6), and conservation of splicing structure, functional domains and motifs, including C-terminal tyrosines (File S4). Phylogenetic analysis identified a distinct third clade related to both *SHP1* and *SHP2*, including a conserved splice site structure that was named SHP3. Analysis of mammalian genomes identified a *SHP3* pseudogene (SHPΨ) with flanking genes (*HES4*, *AGRIN*) showing conserved synteny to zebrafish *shp3* (Figure 6), suggesting selective loss of this *SHP* family member along the mammalian lineage.

### *PIAS* evolution

**Figure 7:**
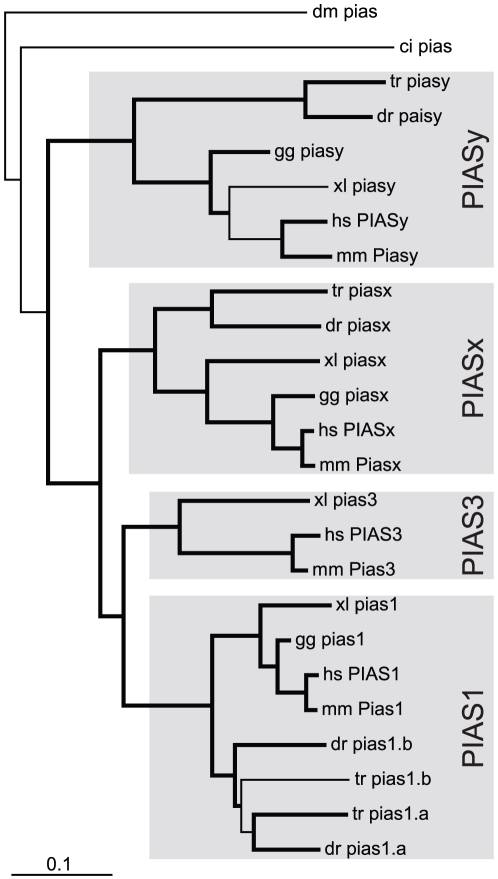
Phylogenetic analysis of *PIAS* family members. Phylogenetic analysis of *PIAS* family members as described for Figure 3.

**Figure 8:**
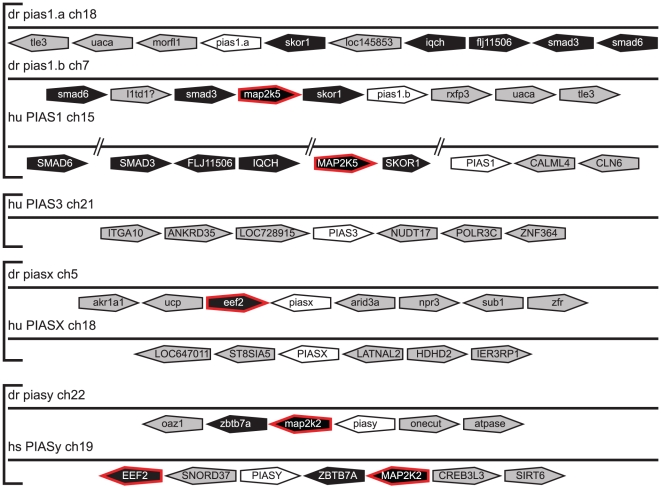
Synteny analysis of *PIAS* family members. Syntenic analysis of *PIAS* family members as described for Figure 4.

There is a single *PIAS* gene in both fruit fly (*pias*) [51] and sea squirt (*pias*) [43], whilst there are four *PIAS* members in mammals (*PIAS1*, *PIAS3*, *PIASx*, *PIASy*) [52]. Bioinformatic analysis revealed the presence of four *pias* genes in zebrafish, the expression of which were confirmed using RT-PCR (Table S1). Phylogenetic analysis indicated that these represented *piasx* and *piasy* orthologs and two *pias1* paralogs, *pias1.a* and *pias1.b*, with no *pias3* ortholog present (Figure 7). The identities of *pias1.a*, *pias1.b* and *piasy* were confirmed by conserved synteny to their mammalian counterparts, including multiple common genes (*SMAD6*, *SMAD3*, *SKOR1*) across both *pias1* genes with additional genes (*IQCH*, *FLJ11506*) or gene (*MAPK* related) for *pias1.a* and *pias1.b*, respectively. There were also two genes (*ZBTB7A*, *MAP2K2*) showing conserved synteny to *piasy*, although there were none for *piasx* between zebrafish and humans (Figure 8). Japanese pufferfish (*Takifugu rubripes*) had the same *pias* complement as zebrafish, whilst African clawed frog (*Xenopus laevis*) possessed the same *pias* complement as mammals, suggesting a teleost-specific absence of *pias3*. Each of the PIAS functional domains, including the SAP, PINIT, RLD, AD, and S/T-rich were conserved in teleost PIAS1 and PIASx homologues. Like their mammalian counterparts [21], teleost PIASy proteins lack both the AD and the S/T-rich region (File S5). Despite extensive searches, additional exons containing the leader sequence for zebrafish *pias1.b* and *piasx* were not found.

### *SOCS* evolution

**Figure 9:**
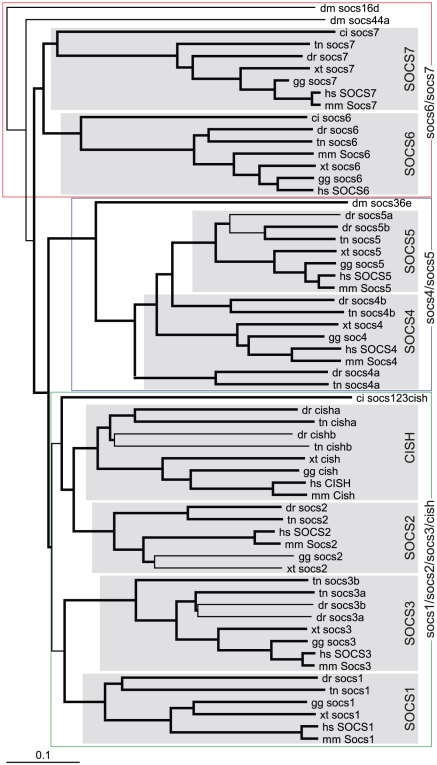
Phylogenetic analysis of *SOCS* family members. Phylogenetic analysis was performed on *SOCS* family members as described for Figure 3.

**Figure 10:**
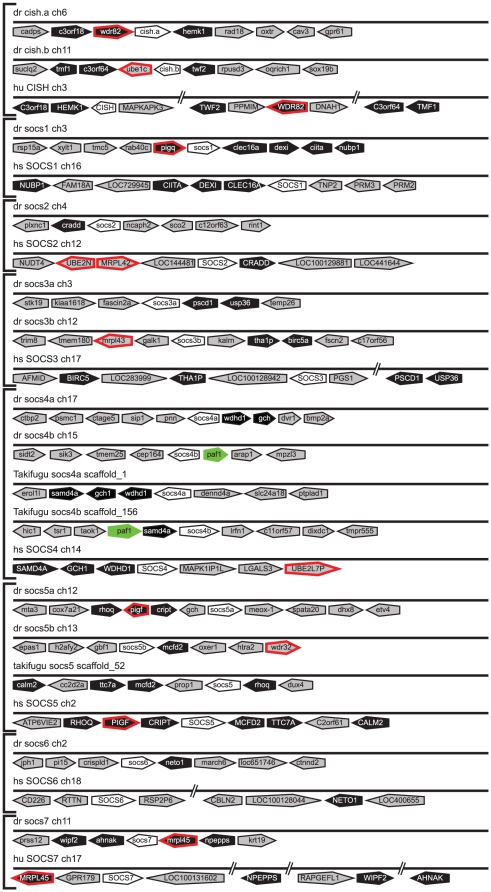
Synteny analysis of *SOCS* family members. Syntenic analysis of *SOCS* family members as described for Figure 4.

In contrast to other JAK-STAT pathway components, which exist as single members in invertebrates, three SOCS family members have been identified in fruit fly (*socs36e, socs44a, socs16d*) [53], [54] as well as in sea squirt (*socs1/socs2/socs3/cish, socs6, socs7*) [43]. The SOCS family has expanded to include 8 members in mammals (*CISH, SOCS1-7*) [55]. Bioinformatic analysis revealed 12 SOCS family members in zebrafish, the expression of which were again confirmed by RT-PCR (Table S1). Phylogenetic analysis identified these as single *socs1*, *socs2*, *socs6 and socs7* orthologs, and paralogous pairs for the remainder: *cisha/cishb*, *socs3a/socs3b*, *socs4a/socs4b*, and *socs5a/socs5b*, with all but *socs5b* also present in pufferfish (Figure 9). The assignments were confirmed by synteny conservation for *cisha* (*C3ORF18, HEMK1, WDR82*) and *cishb* (*TWF2, C3ORF64, TMF1*), *socs1* (*NUBP1, CIITA, DEXI, CLEC16A*), *socs2* (*CRADD*), *socs3a* (*PSCD1, USP36*), *socs3b* (*BIRC5, THA1P*), *socs4a* (*GCH, WDHD1*), *socs5a* (*RHOQ, PIGF, CRIPT*), *socs5b* (*MCFD2, TTC7A, CALM2*), *socs6* (*NETO1*), and *socs7* (*MRPL45, NPEPPS, WIPF2, AHNAK*) (Figure 10). Synteny analysis verified the identity of the teleost *socs4b* by the conserved synteny (*SAMD4A*) between pufferfish *socs4b* and human *SOCS4*, with the identity of zebrafish *socs4b* was confirmed by conserved synteny with pufferfish *socs4b* (*PAF1*). Similar splicing patterns for the zebrafish and mammals SOCS homologues also supported their respective designations (File S6). The SOCS proteins showed conserved domain structure, with the N-terminal regions displaying the lowest degree of sequence conservation, while the SH2 and SOCS box domains were highly conserved between species.

## Discussion

Cytokine receptor signaling is a cornerstone of the immune and hematopoietic systems, with the JAK-STAT pathway representing its major intracellular component. The canonical cytokine receptor-JAK-STAT system, including its key negative regulators, evolved prior to the appearance of chordates, being observed in extant invertebrates such as fruit fly [43]. This study has sought to understand the subsequent evolution and diversification of this system during chordate and vertebrate evolution through the examination of the *JAK*, *STAT*, *SHP*, *PIAS* and *SOCS* families in relevant species. This analysis has identified very limited expansion of these families prior to the divergence of urochordates, but significant expansion from then until the divergence of lobe-finned and ray-finned fishes – coincident with the emergence of adaptive immunity – followed by more moderate expansion from that point. Close examination has provided new insight into the molecular processes involved, the relative pressures for diversification of each signaling component, as well as the overall involvement of cytokine receptor-JAK-STAT pathway in the genesis of the adaptive immune system.

**Figure 11:**
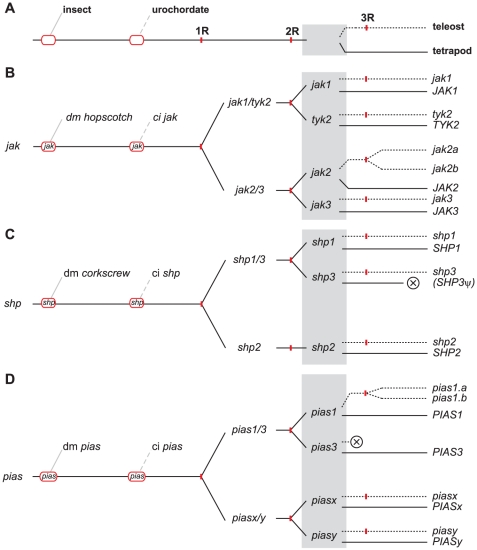
A model for the evolution of the of *JAK*, *SHP*, and *PIAS* families. (A) Evolutionary timeline summarizing key events in chordate/vertebrate evolution. Key evolutionary events are shown, including the divergence of protostomes (solid grey line) from deuterostomes, the subsequent divergence of urochordates (broken grey line) from other chordates, and finally ray-finned fish (including teleosts) (dotted black line) from lobe-finned fish (including terapods) (black line), with relevant present-day groupings (insect, urochordate, teleost, and tetrapod) indicated. Whole genome duplications (1R, 2R, 3R) are indicated with short red lines. (B–D) Model for the evolution of JAK-STAT pathway components: *JAK* (B), *SHP* (C), and *PIAS* (D) families. Red ovals indicate the likely core members at the time of protostomes/deuterostomes and urochordate/chordate divergence, while the grey shaded rectangle represents ray-finned/lobe-finned fish ‘core’ signaling components present at the time of divergence. Lineage-specific/local duplications are indicated by red dots and gene conversion indicated by a red square.

Rather than *de novo* generation of entirely novel genes, gene duplications, domain shuffling, and associated mechanisms play the major role in generating gene diversity within eukaryotes [56]. Gene duplication events can be either local, typically tandem duplications, or global, in the form of whole genome duplication (WGD) events [57], [58]. There have been three WGDs during vertebrate evolution, with the first two (1R and 2R) occurring after the divergence of urochordates but before the divergence of lobe-finned fishes and ray-finned fishes [58], with the third WGD (3R) limited to teleost fish within the ray-finned fish lineage [59] (Figure 11A). These WGDs have led to the so-called ‘1∶2∶4(:8 in fish)’ rule with regards to gene expansion [60]. However, due to a propensity for gene loss as a consequence of insufficient selective pressures following duplication events, this rule generally overestimates the observed level of gene expansion [57], [58]. Gene diversity is also generated by other processes, including the rearrangement of functional domains within a protein through processes such as ‘exon-shuffling’ [61], [62], or specific addition or deletion of specific domains through ‘exonization’ or ‘intronization’, respectively [56]. Our data suggest that WGDs – particularly 1R and 2R – have been the key driver for evolution of JAK-STAT pathway components throughout chordate/vertebrate evolution, with more limited local duplication, and a general paucity of changes in overall domain architecture. Furthermore, positive selection was only detected in a small subset of duplicated members of the signaling pathway following the divergence of lobe-finned and ray-finned fishes (Table S2), suggesting that division of gene function was a largely responsible for gene retention in teleosts and mammals.

The evolution of the *JAK* family follows the classical WGD-driven expansion pattern during 1R and 2R, with one member in invertebrates and urochordates, and four members in tetrapods, including mammals (Figure 11B). This would appear to be via *JAK1/TYK2* and *JAK2/JAK3* intermediates following 1R, as indicated by phylogenetic analysis and conserved synteny across each gene pair. In contrast, only the *JAK2* paralogs, *jak2a* and *jak2b*, were retained in teleost fish following 3R. The evolution of the *SHP* family also appears to have been similarly driven by WGD, although gene retention has been even more limited (Figure 11C). Thus, there is a single homologue in invertebrates and chordates with three members in several higher vertebrates, although only two in mammals. This is most easily explained by 1R generating a *SHP1/SHP3* intermediate and a *SHP2* precursor, with 2R producing separate *SHP1* and *SHP3* genes, but no duplicate retention along the *SHP2* lineage, and with *SHP3* subsequently lost specifically along the mammalian lineage. The additional 3R WGD in teleosts failed to generate any further expansion of *SHP* family members. There has also been no significant change in the domain structure of the proteins encoded by either *JAK* or *SHP* gene families over this evolutionary period. Expansion of the *PIAS* family has also been largely influenced by WGDs (Figure 11D). The 1R event likely generated *PIASx/PIASy* and *PIAS1/PIAS3* intermediates from the single *PIAS* precursor, with 2R generating the individual *PIAS1*, *PIAS3*, *PIASx* and *PIASy* genes. Following 3R the *pias1.a* and *pias1.b* paralogs were retained in the teleost lineage, with the related *pias3* gene being specifically lost. However, unlike the *JAK* and *SHP* families, some limited domain rearrangement was evident in the *PIAS* family, as the sequences encoding the AD and S/T-rich regions were absent specifically within both the mammalian and teleost *PIASy* gene.

**Figure 12:**
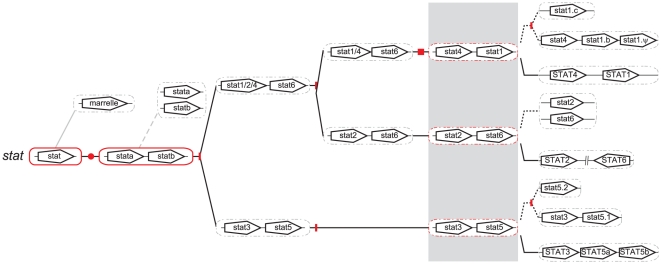
A model for the evolution of the *STAT* family. A model for the evolution of the *STAT* component of the JAK-STAT pathway as described in Figure 11.

The evolution of *STAT* genes has also been influenced by WGD, but significantly supplemented by local duplications, which is emphasized by the proximity of many existing vertebrate *STAT* genes to one another. Indeed, the original *STAT* gene, represented by that in extant invertebrates, was duplicated in a WGD-independent manner by the time of the last common ancestor of urochordates and vertebrates, generating precursors of *stata* and *statb* seen in extant urochordates. A simplistic model that ignored the proximity of *STAT* genes might suggest that the vertebrate *STAT1*, *STAT2*, *STAT3* and *STAT4* genes were generated from the *stata* lineage via classical 1R and 2R WGDs, with *STAT5* and *STAT6* being generated from the *statb* lineage as a result of one of these WGDs. However, an alternative model can be proposed that takes into account the observed proximity within this gene family (Figure 12). This proposes that the *stata* and *statb* precursors originally lay adjacent as a consequence of local duplication, with the *stata-statb* precursor subsequently duplicated *en bloc*, such that 1R and 2R collectively generated four copies of this cluster, only three of which were retained: a *STAT3*-*STAT5* cluster, a *STAT2*-*STAT6* cluster, and a *STAT1-STAT4* cluster. In support of this model, the first two of these clusters maintain the appropriate *stata-statb* precursor configuration. The latter cluster consists of two *stata*-related genes, although this arrangement can be explained through sequential gene loss (of the *statb* equivalent) followed by local duplication of the remaining gene, or via ‘gene conversion’ of the adjacent *STAT* genes, as has been reported for a segment of the adjacent mammalian *STAT5* genes [63]. Analysis of additional organisms intermediate between urochordates and higher vertebrates will definitively resolve this issue. Finally, duplicates for both *stat1* and *stat5* have been retained following 3R in teleosts, with additional local duplications of *stat1* leading to a pseudogene in zebrafish, while local duplication has also occurred along the mammalian lineage with respect to the *STAT5* genes.

**Figure 13:**
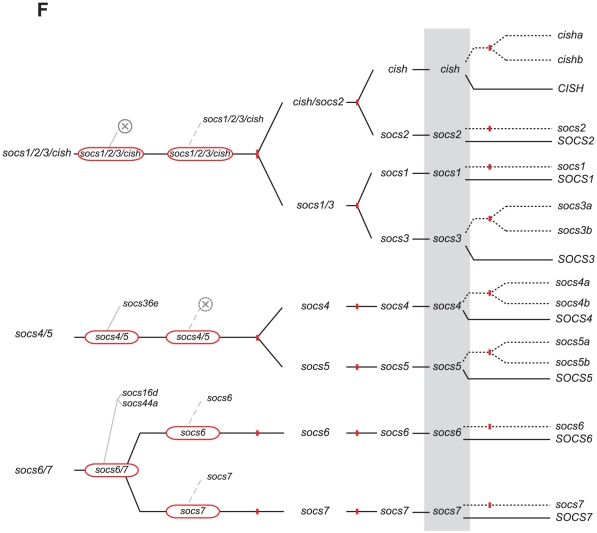
A model for the evolution of the *SOCS* family. A model for the evolution of the *SOCS* component of the JAK-STAT pathway as described in Figure 11.

Interpretation of *SOCS* gene evolution is also complex, since certain gene subsets appear to have been specifically lost in some lineages. Our model suggests that the common ancestor of protostomes and deuterostomes possessed four members of this family: a *socs1/socs2/socs3/cish* intermediate, a *socs4/socs5* intermediate as well as distinct *socs6* and *socs7* precursors (Figure 13). This is supported by analysis of the more primitive sea anemone (*Nematostella vectensis*) genome, which contains exactly this complement of *socs* genes (data not shown). Additionally, a *SOCS2-like* gene, resembling the *socs1/socs2/socs3/cish* intermediate, has been described in the European honey bee (*Apis mellifera*), flour beetle (*Tribolium castaneum*), along with various molluscs and crustaceans [64]. Thus, the invertebrate lineage of protostomes appears to possess a *socs1/socs2/socs3/cish* intermediate, lost in the fruit fly, whilst protostomes also possess a *socs4/socs5* intermediate, as well as divergent *socs6* and *socs7* orthologs, whereas the urochordate lineage of deuterostomes (typified by sea squirt) has retained the *socs1/socs2/socs3/cish* intermediate, as well as *socs6* and *socs7* orthologs, but has specifically lost the *socs4/socs5* intermediate. Subsequent expansion of the *SOCS* family within vertebrates has been variable along the different lineages, although WGDs again represent the key driving force. The *SOCS1/SOCS2/SOCS3/CISH* lineage follows the classical WGD expansion during 1R and 2R, generating *SOCS1*, *SOCS2*, *SOCS3* and *CISH*, via *SOCS1/SOCS3* and *SOCS2/CISH* intermediates, with additional *socs3* and *cish* paralogs retained in teleosts following 3R. The *SOCS4/SOCS5* intermediate has only duplicated once during 1R and 2R, with the presence of two distinct *socs4*/*socs5* genes in the genome of *Petromyzon marinus* (data not shown) suggesting that the duplication occurred via 1R. The 3R event produced paralogs for both *socs4* and *socs5* that have been retained in zebrafish. In contrast, no expansion of *socs6* or *socs7* genes has occurred following any of the three WGD events.

From this analysis it is evident that diversification of individual JAK-STAT pathway components has occurred at differing rates. For 1R and 2R, the overall ‘expansion ratio’ (calculated by comparing the number of members present in the common ancestor of protostomes and deuterostomes to the common ancestor of tetrapods and teleosts) was 1∶4 for the *JAK* and *PIAS* families and 1∶3 for the *STAT* and *SHP*. The expansion ratio for the *SOCS* family overall was 1∶2, although this ranged markedly between sub-families: from 1∶4 for *SOCS1/SOCS2/SOCS3/CISH*, 1∶2 for *SOCS4/SOCS5*, and 1∶1 for *SOCS6* and *SOCS7*. For 3R, the expansion was more modest and differentially focused, being 1∶1.5 for the *SOCS* family, 1∶1.33 for the *STAT* family, 1∶1.25 for the *JAK* family and 1∶1 for the *SHP* and *PIAS* families. Indeed, the majority of *JAK-STAT* pathway components are represented at a 1∶1 homolog ratio between tetrapods and teleosts. The encoded set of core signaling molecules (JAK1, JAK3, TYK2, STAT2, STAT3, STAT4, STAT6, SOCS1, SOCS2, SOCS6, SOCS7, SHP1, SHP2, PIASx, PIASy) are therefore likely to display the highest functional conservation across these organismal groups.

The differential expansion of the individual JAK-STAT pathway components would not seem to be a random process, but instead linked to specific signaling “modules”. The key factor appears to be the diversification of upstream cytokine receptors, which expanded >30-fold during 1R and 2R but with much more limited expansion during 3R [65], [66], [67], consistent with the relative expansion of JAK-STAT pathway components. Specific evidence for the role of cytokine receptor expansion in the process comes from analysis of the *SOCS* family, with the subset predominantly involved in regulating cytokine signaling (*SOCS1*, *SOCS2*, *SOCS3*, *CISH*) expanding 4-fold during 1R and 2R, the subset with lesser roles (*SOCS4*, *SOCS5*) expanding to a reduced extent, and the subset with no known role in cytokine signaling (*SOCS6*, *SOCS7*) not expanding at all. Duplicate retention of *JAK-STAT* components along the teleost lineage (3R) also strongly correlates with expansion of the corresponding cytokine receptors, such that entire pathways are seen to be replicated. For example, duplicates of both *prolactin receptor (prlr)* and *growth hormone receptor (ghr)* are found in teleosts [67], as are the genes encoding the JAK-STAT components lying downstream of these receptors (*JAK2*, *STAT5*, *CISH*). Similarly, Class II receptors have expanded along the teleost lineage [65], [68], [69], as have the genes encoding *STAT1* and its specific negative regulator *PIAS1*, that lie downstream of this group of receptors [70]. Interestingly, the expansion of cytokine receptors has significantly exceeded that of the downstream JAK-STAT pathway. However, the latter are pleiomorphic, being able to form distinct ‘signaling modules’ by combining different components. For example, JAK2 can differentially activate STAT1, STAT3, STAT6 and/or STAT6 depending on the receptor context [7], while negative regulators are able to act on multiple receptors, JAKs and STATs [8], including the ability to cross-talk between different receptors [71]. Therefore the overall functional complexity of both extracellular and intracellular signaling has probably increased to a similar extent. Furthermore, the relative rates of evolution for JAK-STAT components is different for those which are largely immune restricted (JAK3, TYK2, STAT1, STAT2, STAT4, and STAT6) compared to those that are more pleiotropic (JAK1, JAK2, STAT3, and STAT5) (Table S2). Consistent with a previous study [72], the higher dN/dS ratios of the immune restricted components reflect a greater evolutionary rate of change and lower purifying selection than the more pleiotropic components, likely due to the constant need to respond to the ever changing pathogenic threats that the immune system encounters.

Finally, this study provides evidence that diversification of cytokine receptor signaling through JAK-STAT pathway components has contributed to the emergence of the adaptive immune system (AIS). The AIS arose within a relatively short time interval in vertebrate evolution, in a so-called immunological ‘big bang’ [73] associated with two whole genome duplications [74]. Whilst this likely overstates the simplicity and rapidity involved [75], [76], the WGD events clearly provided much of the raw materials for specific aspects of adaptive immunity [77], such that genes involved in adaptive immunity have been shown to be preferentially retained following 1R and 2R [69]. This is largely true for all JAK-STAT components, with the exception of the subfamily of SOCS proteins with roles outside of cytokine signaling and SHP proteins that also participates in growth factor signaling [78] thereby limiting the potential for additional diversification of this family. Moreover, the upstream cytokine receptors have been massively diversified in this time period and seem a major driver for retention of downstream JAK-STAT components, including those with unique roles in adaptive immunity. Indeed, the core cytokine signaling pathway components involved in adaptive immunity are present following 1R and 2R [65], [66], [67], including the lymphocyte-specific IL-2R, IL-4R, JAK3, STAT4 and STAT6, as are the key AIS components [76]. As a corollary, the additional diversification during 3R falls largely outside the immune system (eg. PRLR/GHR pathways and SOCS4/SOCS5), apart from some limited diversification of class II cytokine signaling along the teleost lineage [65]. In contrast, the innate immune system, typified by the presence of immune recognition molecules and phagocytic cells, pre-dates the evolution of functional cytokine receptor signaling. For example, Purple sea urchin (*Strongylocentrotus purpuratus*), possesses over 200 Toll-like receptors (TLRs), but no apparent cytokine receptor signaling system [79]. Similarly, fruit fly has several distinct innate immune cell populations and defense systems, but its canonical cytokine receptor-JAK-STAT pathway has only limited roles within its immune system [22]. Rather, the subsequent diversification of cytokine receptor-JAK-STAT pathways would appear to contribute to the refinement of the pre-existing innate immune system. This is illustrated, for example, by the G-CSFR that is not essential for the development of innate immune cells, but also allows the innate immune system to respond to ‘emergency’ situations [4].

### Conclusions

The canonical cytokine receptor-regulated JAK-STAT pathway was formed prior to the appearance of chordates and has subsequently diversified greatly during chordate and vertebrate evolution. This significant, but differential, expansion of pathway components is largely mediated by WGD events with retention driven by diversification of upstream cytokine receptors. The majority of this occurred co-incidentally with the appearance of adaptive immunity, at which time the key lymphoid-specific cytokine signaling pathways were generated. This collectively suggests that evolution of cytokine receptor signaling via the JAK-STAT pathway was a key facilitator of adaptive immune system emergence.

## Methods

### Genomic data mining and sequence analysis

The tBLASTn algorithm was employed to systematically search for *JAK*, *STAT*, *SHP*, *PIAS* and *SOCS* gene sequences in Expressed Sequence Tag (EST), genomic, and whole genome shotgun (WGS) databases for a range of organisms at GenBank (http://blast.ncbi.nlm.nih.gov/), or in other specific genomic databases, such as sea squirt (http://genome.jgi-psf.org/ciona4/). All independent sequences identified that possessed E values<0.1 were extracted for further analysis. GenomeScan (http://genes.mit.edu/genomescan.html) was used to predict coding exons from sequences derived solely from WGS or genomic scaffolds, some of which were manually adjusted on the basis of known intron-exon boundaries in other organisms. Nucleotide sequences were assembled using Sequencher 4.1.4 (Gene Codes Corporation). Any apparently incomplete contigs were extended by iterative BLASTn searches using the relevant contig terminus until the entire putative coding sequence had been identified. Any remaining gaps in the contigs were closed by sequencing of appropriate reverse transcription-polymerase chain reaction (RT-PCR) products. The probable identity of each encoded protein was determined by BLASTp searching with the respective conceptual translations. To analyze splicing the position of intron/exon boundaries was determined by alignment of cDNA and genomic sequences, applying the GT-AG splice rule where possible [80]. The final assignment of identity was guided by overall sequence identity, conservation of key functional domains and residues, phylogenic analysis and conserved synteny. The nomenclature for the genes followed the conventions of GenBank and the zebrafish information network (ZFIN) (www.zfin.org).

### Phylogenetic analysis

Multiple protein sequences were aligned using AlignX9 (Invitrogen) and ClustalX 1.83 [81]. The latter was utilized to create bootstrapped phylogenetic trees of 1000 replicates using the Neighbor-Joining algorithm, with trees formatted using Njplot (http://pbil.univ-lyon1.fr/software/njplot.html), and viewed in Treeview 1.6.6 (http://taxonomy.zoology.gla.ac.uk/rod/treeview.html). Additional analyses using Maximum parsimony and Maximum likelihood algorithms were performed with Phylo_win (http://pbil.univ-lyon1.fr/software/phylowin.html) and Phylip (http://evolution.genetics.washington.edu/phylip.html) packages to confirm phylogenetic topologies. The JCoDA software package (http://www.tcnj.edu/~nayaklab/jcoda) was used to calculate positive selection.

### Synteny analysis

Ensembl (http://www.ensembl.org) was used to perform synteny analysis using the following genome assemblies: sea squirt (*Ciona intestinalis*) (JGI 2), zebrafish (*Danio rerio*) (Zv6 and Zv7), spotted green pufferfish (*Tetraodon nigroviridis*) (TETRAODON 7), Japanese pufferfish (*Takifugu rubripes*) (FUGU 4.0), African clawed frog (*Xenopus tropicalis*) (JGI 4.1), chicken (*Gallus gallus*) (WASHUC 1), mouse (*Mus musculus*) (NCBI m36) and human (*Homo sapiens*) (NCBI 36).

## Supporting Information

Table S1 **Homology and expression analyses for the JAK, STAT, SHP, PIAS and SOCS families.** Zebrafish homologues for the JAK, STAT, SHP, PIAS and SOCS families are listed along with the human homologues and conserved synteny indicated. Expression was confirmed by detection of an appropriately-sized RT-PCR product following agarose gel electrophoresis, or from previous publications.
(DOC)

Table S2 **Positive selection for the JAK, STAT, SHP, PIAS and SOCS families.** Zebrafish, mouse and humans genes were compared with duplicates combined into the same calculation for positive selection. The pairwise score (dN/dS) for each gene set was averaged. The M7 and M8 models were compared for the likelihood ratio rest and were bolded if p<0.05, thus indicating positive selection. Duplicated zebrafish genes are indicated by an asterisk (*).
(DOC)

File S1 **Strategy for the identification and characterization of JAK-STAT pathway genes.** Flowchart of the three components of the identification and characterisation strategy: (i) sequence search, involving database interrogation, sequence assembly and prediction (red boxes), (ii) sequence identification and confirmation, involving sequence alignment, phylogenetic analysis, conserved domain/motif confirmation, and synteny analysis (blue boxes), collectively generated a candidate homologue (green box) for subsequent (iii) expression analysis, via RT-PCR (yellow boxes).
(EPS)

File S2 **Splice site and domain analysis of the** ***JAK*** **family.** Analysis of splice sites structure within the *JAK* gene family in zebrafish (dr) and human (hs). Exons are indicated as squares, with introns shown as open triangles. Specific domains within each protein family are shaded and labeled, with essential tyrosine motifs for JAK proteins indicated by broken black lines and the STAT2 KYLK motif shown by a broken white line.
(EPS)

File S3 **Splice site and domain analysis of the** ***STAT*** **family.** Analysis of splice sites structure within the *STAT* gene family as described in File S2 with the addition of sea squirt (ci) and the STAT2 KYLK motif shown by a broken white line.
(EPS)

File S4 **Splice site and domain analysis of the** ***SHP*** **family.** Analysis of splice sites structure within the *SHP* gene family as described in File S2 with essential tyrosine motifs for SHP proteins indicated by broken black lines.
(EPS)

File S5 **Splice site and domain analysis of the** ***PIAS*** **family.** Analysis of splice sites structure within the *PIAS* gene family as described in File S2.
(EPS)

File S6 **Splice site and domain analysis of the** ***SOC*** **family.** Analysis of splice sites structure within the *SOC* gene family as described in File S2.
(EPS)
