# Supplementary material for: Evolution of JAK-STAT Pathway Components: Mechanisms and Role in Immune System Development
Source: PLoS One. 2012 Mar 7;7(3):e32777. doi: 10.1371/journal.pone.0032777 (PMC3296744; doi:10.1371/journal.pone.0032777)
Supplement: Table S1 — Homology and expression analyses for the JAK, STAT, SHP, PIAS and SOCS families. Zebrafish homologues for the JAK, STAT, SHP, PIAS and SOCS families are listed along with the human homologues and conserved synteny indicated. Expression was confirmed by detection of an appropriately-sized RT-PCR product following agarose gel electrophoresis, or from previous publications. (DOC) [file pone.0032777.s001.doc]

| **Family** | **Zebrafish sequence** | **Human homology** | | **Expression detected** | **Reference** |
| --- | --- | --- | --- | --- | --- |
|  |  | **Most similar** | **Synteny** |  |  |
| ***JAK*** | *jak1* | *JAK1* | Yes | Yes | (28) |
|  | *jak2a* | *JAK2* | Yes | Yes | (29) |
|  | *jak2b* | *JAK2* | Yes | Yes | This study |
|  | *jak3* | *JAK3* | Yes | Yes | This study |
|  | *tyk2* | *TYK2* | Yes | Yes | This study |
| ***STAT*** | *stat1.a* | *STAT1* | Yes | Yes | (31) |
|  | *stat1.b* | *STAT1* | Yes | Yes | This study |
|  | *stat1ψ* | *STAT1* | Yes | Yes | This study |
|  | *stat2* | *STAT2* | No | Yes | This study |
|  | *stat3* | *STAT3* | Yes | Yes | (31) |
|  | *stat4* | *STAT4* | Yes | Yes | This study |
|  | *stat5.1* | *STAT5* | Yes | Yes | (32) |
|  | *stat5.2* | *STAT5* | Yes | Yes | (32) |
|  | *stat6* | *STAT6* | Yes | Yes | This study |
| ***SHP*** | *shp1* | *SHP1* | Yes | Yes | This study |
|  | *shp2* | *SHP2* | Yes | Yes | This study |
|  | *shp3* | *SHP3ψ* | Yes | Yes | This study |
| ***PIAS*** | *pias1.a* | *PIAS1* | Yes | Yes | This study |
|  | *pias1.b* | *PIAS1* | Yes | Yes | This study |
|  | *piasx* | *PIASx* | No | Yes | This study |
|  | *piasy* | *PIASy* | Yes | Yes | This study |
| ***SOCS*** | *cish.a* | *CISH* | Yes | Yes | This study. |
|  | *cish.b* | *CISH* | Yes | Yes | This study |
|  | *socs1* | *SOCS1* | Yes | Yes | This study |
|  | *socs2* | *SOCS2* | Yes | Yes | This study |
|  | *socs3a* | *SOCS3* | Yes | Yes | This study |
|  | *socs3b* | *SOCS3* | Yes | Yes | This study |
|  | *socs4a* | *SOCS4* | Yes | Yes | This study |
|  | *socs4b* | *SOCS4* | No# | Yes | This study |
|  | *socs5a* | *SOCS5* | Yes | Yes | This study |
|  | *socs5b* | *SOCS5* | Yes | Yes | This study |
|  | *socs6* | *SOCS6* | Yes | Yes | This study |
|  | *socs7* | *SOCS7* | Yes | Yes | This study |

# although conserved synteny was not observed between humans and zebrafish, further analysis showed conserved synteny between Japanese pufferfish and humans.
