# Supplementary material for: Evolution of JAK-STAT Pathway Components: Mechanisms and Role in Immune System Development
Source: PLoS One. 2012 Mar 7;7(3):e32777. doi: 10.1371/journal.pone.0032777 (PMC3296744; doi:10.1371/journal.pone.0032777)
Supplement: Table S2 — Positive selection for the JAK, STAT, SHP, PIAS and SOCS families. Zebrafish, mouse and humans genes were compared with duplicates combined into the same calculation for positive selection. The pairwise score (dN/dS) for each gene set was averaged. The M7 and M8 models were compared for the likelihood ratio rest and were bolded if p<0.05, thus indicating positive selection. Duplicated zebrafish genes are indicated by an asterisk (*). (DOC) [file pone.0032777.s002.doc]

| Family | Gene | dN/dS | M7 vs M8 |
| --- | --- | --- | --- |
| **JAK** | JAK1 | 0.06 | 4.09 |
|  | JAK2* | 0.05 | 0.08 |
|  | JAK3 | 0.08 | **11.33** |
|  | TYK2 | 0.10 | 4.34×10^-04^ |
| **STAT** | STAT1* | 0.14 | **8.17** |
|  | STAT2 | 0.28 | **18.85** |
|  | STAT3 | 0.01 | 1.05 |
|  | STAT4 | 0.06 | 4.56×10^-04^ |
|  | STAT5* | 0.03 | 2.48 |
|  | STAT6 | 0.14 | 2.60×10^-04^ |
| **SHP** | SHP1 | 0.05 | 4.92×10^-03^ |
|  | SHP2 | 0.02 | 5.57×10^-03^ |
|  | SHP3 | - | - |
| **PIAS** | PIAS1* | 0.05 | 0.50 |
|  | PIAS3 | - | - |
|  | PIASx | 0.06 | 8.24×10^-03^ |
|  | PIASy | 0.06 | 0.39 |
| **SOCS** | CISH* | 0.24 | 4.60×10^-05^ |
|  | SOCS1 | 0.11 | 1.95×10^-03^ |
|  | SOCS2 | 0.06 | 2.09 |
|  | SOCS3* | 0.06 | 2.86×10^-04^ |
|  | SOCS4* | 0.12 | **6.06** |
|  | SOCS5* | 0.09 | 4.37 |
|  | SOCS6 | 0.10 | **15.00** |
|  | SOCS7 | 0.14 | 7.11×10^-03^ |
